# Effects of the gut microbiota and its metabolite short-chain fatty acids on endometriosis

**DOI:** 10.3389/fcimb.2024.1373004

**Published:** 2024-06-13

**Authors:** Menghe Liu, Ru Peng, Chunfang Tian, Jianping Shi, Jiannan Ma, Ruiwen Shi, Xiao Qi, Rongwei Zhao, Haibin Guan

**Affiliations:** ^1^ College of Pharmacy, Inner Mongolia Medical University, Hohhot, Inner Mongolia Autonomous Region, China; ^2^ Department of Obstetrics and Gynecology, Hohhot Maternal and Child Health Care Hospital, Hohhot, Inner Mongolia Autonomous Region, China; ^3^ Department of Oncology, Inner Mongolia Traditional Chinese Medicine Hospital, Hohhot, Inner Mongolia Autonomous Region, China; ^4^ College of Traditional Chinese Medicine, Inner Mongolia Medical University, Hohhot, Inner Mongolia Autonomous Region, China; ^5^ Department of Obstetrics and Gynecology, Affiliated Hospital of Inner Mongolia Medical University, Hohhot, Inner Mongolia Autonomous Region, China

**Keywords:** gut microbiota, short-chain fatty acids, endometriosis, mechanism of action, treatment strategy

## Abstract

In recent years, a growing body of research has confirmed that the gut microbiota plays a major role in the maintenance of human health and disease. A gut microbiota imbalance can lead to the development of many diseases, such as pregnancy complications, adverse pregnancy outcomes, polycystic ovary syndrome, endometriosis, and cancer. Short-chain fatty acids are metabolites of specific intestinal bacteria and are crucial for maintaining intestinal homeostasis and regulating metabolism and immunity. Endometriosis is the result of cell proliferation, escape from immune surveillance, and invasive metastasis. There is a strong correlation between the anti-proliferative and anti-inflammatory effects of short-chain fatty acids produced by gut microbes and the development of endometriosis. Given that the mechanism of action of gut microbiota and Short-chain fatty acids in endometriosis remain unclear, this paper aims to provide a comprehensive review of the complex interactions between intestinal flora, short-chain fatty acids and endometriosis. In addition, we explored potential microbial-based treatment strategies for endometriosis, providing new insights into the future development of diagnostic tests and prevention and treatment methods for endometriosis.

## 1 Introduction

Endometriosis (EMS) is a disease of the endometrium or endometrial tissue that is active outside the uterine cavity, such as in the peritoneal cavity, ovary, bladder or ureter, or even in the distal organs (Smolarz et al., 2021). The growth of endometrial implants and associated inflammation lead to increased production of pro-inflammatory cytokines (Tai et al., 2018). These processes eventually lead to chronic inflammation, the formation of pelvic adhesions and EMS, which impairs reproductive function (Jiang et al., 2021). In addition to gynecological symptoms, up to 90% of EMS patients experience gastrointestinal symptoms (Bloski and Pierson, 2008; Maroun et al., 2009; DiVasta et al., 2018). The researchers found that the human gut microbiota (GM) and the development of EMS are bidirectional (Jiang et al., 2021). The human gut microbiome is composed of bacteria, fungi, protozoa, archaea and some viruses, whose encoding genes are approximately 150 times the total number of human genes; bacteria play a dominant role and include *Firmicutes*, *Bacteroidetes*, *Proteobacteria* and *Actinobacteria*, four key bacteria (Senchukova, 2023). The type and quantity of the gut microbiota (GM) are affected by the environment, diet, genetics, drugs, disease and other factors. Studies have shown that the gut microbiota plays an important role in various kinds of inflammation (Qin et al., 2022). A study in monkeys found that monkeys with endometriosis had lower levels of *lactobacillus* and higher levels of gram-negative bacteria, and a higher incidence of intestinal inflammation than healthy controls (Bailey and Coe, 2002). Another study showed that mice with endometriosis showed a general decrease in *bacteroides* levels (Yuan et al., 2018). The mechanism by which gut microbiota affects EMS may involve estrogen metabolism, immune dysregulation and inflammatory response (Hantschel et al., 2019; Salliss et al., 2021).

In addition, one mechanism by which mammalian gut bacteria influence host physiological and immune processes is fermentation to produce hundreds of metabolites that regulate key host functions, including short-chain fatty acids (SCFAs) such as butyrate, propionate, and acetate, which are involved in maintaining intestinal barrier function, improving host metabolism, and regulating the immune system and inflammatory response (Martin-Gallausiaux et al., 2021). SCFAs is used by intestinal cells as an energy source or transported into the bloodstream and can have anti-proliferative and anti-inflammatory effects on distant organs (Maslowski et al., 2009; den Besten et al., 2013; Semaan et al., 2020). For example, n-butyrate inhibits the proliferation of colorectal cancer cells (Cao et al., 2019). N-butyrate can inhibit the expression of pro-inflammatory cytokines tumor necrosis factor α (TNF-α) and IL-6 in lipopolysaccharide-induced macrophages (Chen et al., 2018). Chadchan et al. reported that SCFAs can inhibit the growth of EMS ectopic lesions (Chadchan et al., 2021). SCFAs exerts anti-inflammatory and regulatory effects on immune cells mainly by activating G-protein-coupled receptors GPR43, GPR41 and GPR109A and inhibiting histone deacetylase, thereby inhibiting the development of EMS (Licciardi et al., 2011; Smith et al., 2013; Park et al., 2015). Future studies on the functional characterization of the microbiome will greatly assist in the development of microbiome-based therapeutics for the treatment of EMS. This review conducted a computer search for articles related to the gut microbiota, short-chain fatty acids and the endometriosis published in the PubMed (https://pubmed.ncbi.nlm.nih.gov/advanced/), Medline databases (https://ovidsp.ovid.com/autologin.cgi), Wan Fang Data Knowledge Service Platform (https://www.wanfangdata.com.cn), CNKI (https://www.cnki.net) and China Biomedical Literature Service System (http://www.sinomed.ac.cn/zh/). The search time limit was from the establishment of each database until March 2024. This paper describes the specific mechanisms involved and the future clinical application of the gut microbiota and its metabolites SCFAs in affecting EMS.

## 2 Endometriosis

EMS is a chronic inflammatory gynecological disease characterized by abnormal growth of the endometrial epithelium or stroma outside the uterus (Zondervan et al., 2018). Endometriosis often occurs in the pelvic area, including the ovaries, fallopian tubes, peritoneal surface, bladder, rectovaginal septum, and gastrointestinal tract. Distal organ endometriosis has also been reported, such as liver EMS, brain EMS, and chest EMS (da Costa et al., 2022; Elefante et al., 2022; Topbas Selcuki et al., 2022; Posses Bridi et al., 2023). The symptoms of EMS are often pelvic pain, dysmenorrhea, and infertility (Zondervan et al., 2018). The incidence of EMS in women of reproductive age is as high as 10% globally, seriously affecting the physical health of patients, and causing a huge economic burden (Simoens et al., 2012; Zondervan et al., 2020). EMS is a multifactorial disease whose pathogenesis has not been fully elucidated, and many factors have been scientifically investigated (Burney and Giudice, 2012). The most widely accepted pathogenesis of EMS is the theory of menstruation retrogression, in which endometrial tissue returns to the pelvic cavity through the fallopian tube during menstruation, and after reaching the pelvic cavity, endometrial stromal cells adhere to the peritoneal surface and proliferate, developing into invasive lesions (Halme et al., 1984; Yovich et al., 2020). Tai et al. suggested a link between EMS and pelvic infection. They included data from more than 14,000 patients to assess the association between pelvic inflammatory disease and endometriosis, showing a three-fold increased risk of EMS in patients with pelvic inflammatory disease (Tai et al., 2018). A growing number of studies have found links between EMS and chronic as well as autoimmune diseases (Kvaskoff et al., 2015; Zhang et al., 2018). Studies of the occurrence of endometriosis in identical twins suggest that EMS has a genetic basis (Hadfield et al., 1997; Hansen and Eyster, 2010). In addition, low birth weight, earlier age of menarche, and shorter menstrual cycles were all found to be significantly associated with a higher risk of EMS (Matalliotakis et al., 2008; Nnoaham et al., 2012; Borghese et al., 2015). There are many other indirect risk factors for EMS, including diet (e.g., alcohol and caffeine intake), environment (e.g., polychlorinated biphenyl and dioxin), and lifestyle (e.g., night shift work), but further research is needed to find a definitive association (Rier et al., 1993; Hemmings et al., 2004; Chiaffarino et al., 2014; Smarr et al., 2016; Jurkiewicz-Przondziono et al., 2017; Shafrir et al., 2018).

Currently, EMS is mainly confirmed through invasive procedures such as laparoscopy and histopathology EMS (Agarwal et al., 2019). Because EMS is often associated with gynecological, pain, and gastrointestinal disorders, its non-specific manifestations and highly limited diagnostic methods lead to delayed diagnosis and treatment of EMS (Arruda et al., 2003; McGrath et al., 2023). Conventional treatment with EMS (such as surgery and hormone therapy) can relieve associated symptoms (Bedaiwy et al., 2017). However, the recurrence rate is high, and there are side effects of hormone therapy and surgical risks (Vercellini et al., 2008). Therefore, it is necessary to search for non-invasive biomarkers to diagnose and treat EMS. It has been reported that some diagnostic markers in the urine, serum, menstrual blood, and other body fluids of women with endometriosis can circumvent unnecessary invasive techniques and have the advantages of being simple, rapid, and non-invasive (Anastasiu et al., 2020). Therefore, biomarkers have become an important direction in the diagnosis of this disease. There is a close relationship between gastrointestinal and reproductive tract microecology and EMS (Sobstyl et al., 2023). One study showed that the vaginal microbiome is less important as a predictor than the gut microbiome in diagnosing endometriosis (Huang et al., 2021b). This brings a new idea for the research of EMS. Recent studies have shown that intestinal microbiota and its small molecule metabolite SCFAs can affect the occurrence and development of EMS, which is expected to become a new biomarker of EMS (Chadchan et al., 2021).

## 3 Gut microbiota metabolites: SCFAs

**Figure 1:**
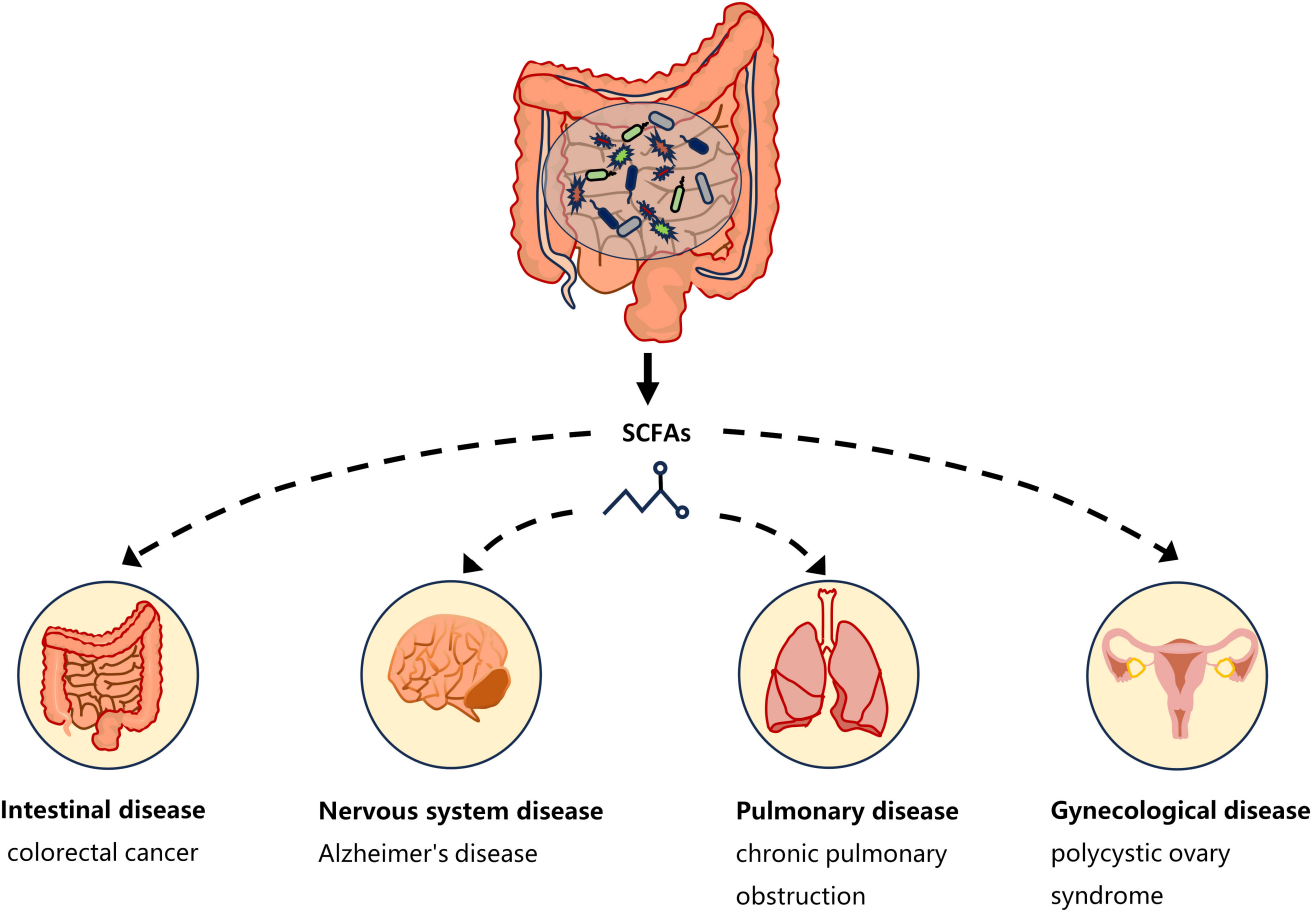
SCFAs and disease types. Short-chain fatty acids produced by gut microbiota have a complex relationship with intestinal diseases (such as colorectal cancer), neurological diseases (such as Alzheimer**’**s disease), gynecological diseases (such as polycystic ovary syndrome), and lung diseases (such as chronic pulmonary obstruction). The continuous lines indicate the direct action, and the fragmented lines indicate the indirect action.

One of the ways in which the gut microbiota affects host physiology may be through the production of the metabolite SCFAs. The critical role of microbiota in the production of SCFAs has been reported in a comparison of germ-free and normal mice (Høverstad and Midtvedt, 1986). SCFAs are saturated fatty acids with no more than six carbon atoms that are produced by specific bacteria in the cecum and colon through the fermentation of undigested dietary fiber (Cong et al., 2022). The main SCFAs in the intestine are acetate, propionate and butyrate, accounting for more than 95% of all SCFAs (Cummings et al., 1987). SCFAs is not only present in the gut, but can also be transported through the gut into the blood circulation (Pomare et al., 1985). Studies have shown that changes in SCFAs concentrations in the blood or tissues can cause inflammatory, immune, and metabolic diseases (Morris et al., 2017). SCFAs affects inflammation and immunity by regulating the production of immune mediators, cytokines and chemokines, as well as the differentiation, recruitment and activation of immune cells such as neutrophils, macrophages, DC and T lymphocytes (Corrêa-Oliveira et al., 2016). A growing body of evidence highlights the role of SCFAs in the gut as well as in the tissues and organs outside the gut (**Figure 1**). Low levels of butyrate producing bacteria and short-chain fatty acids have been found in patients with colorectal cancer (Wang et al., 2019). High levels of propionate in the blood prevent allergic inflammation in the lungs by enhancing the production of macrophages and DC precursors by GPR4 (Trompette et al., 2014). *Clostridium butyricum* may reduce microglia-mediated neuroinflammation in Alzheimer’s disease by regulating the metabolite butyrate (Sun et al., 2020). In addition, recent studies have found a complex relationship between SCFAs and EMS. Metabolomics showed reduced short-chain fatty acids in a mouse model of endometriosis (Alghetaa et al., 2023). Studies by Chadchan et al. also found that short-chain fatty acids were reduced in the feces of mice with endometriosis, and treatment with n-butyrate reduced the growth of endometriosis lesions in mice (Chadchan et al., 2021). A case-control study of 137 women found that the serum metabolite 2-hydroxybutyric acid was associated with endometriosis risk (Lefebvre et al., 2024). These studies have proved that SCFAs can affect the occurrence and development of EMS.

## 4 Relationship between gut microbiota imbalance and EMS

**Figure 2:**
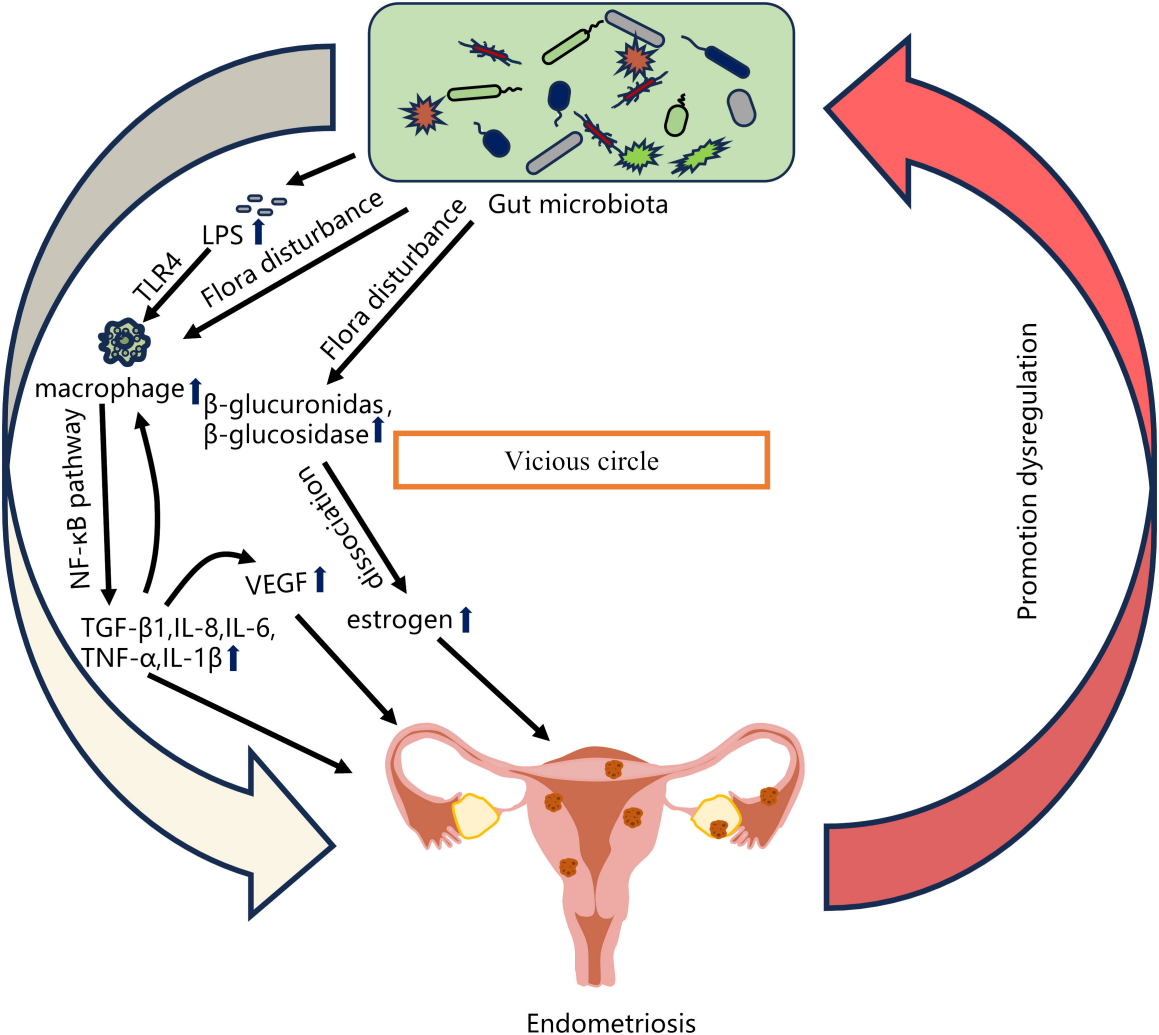
Relationship between gut microbiota imbalance and EMS. Possible explanations for the effect of gut microbiota dysbiosis on endometriosis include the bacterial contamination theory (Dysregulation of the gut microbiota leads to increased LPS levels, and LPS-induced primary inflammation promotes the secretion of the NF-κB mediator in the peritoneal cavity through TLR4 present on macrophages and other immune cells, and produces significant levels of TNF-α and IL-8), estrogen metabolism (When intestinal microbiota is disturbed, β-glucuronidase and β-glucosidase secreted by estrogen group can degrade bound estrogen, resulting in excessive free estrogen), cytokines and impaired immune function (The imbalance of intestinal microbiota leads to the increase of pro-inflammatory cytokines such as TGF-β1, IL-8, IL-6, TNF-α and IL-1β. The local surge of cytokines further promotes the recruitment of immune cells and the secretion of more cytokines, and stimulates the production of VEGF, thereby inducing endometrial cell proliferation). EMS long-term inflammation will further promote intestinal microbiota imbalance, thus forming a vicious cycle.

A number of studies have shown that changes in the gut microbiota are associated with the pathogenesis of EMS (**Table 1**). Chadchan et al. gavaged metronidazole-treated mice with the feces of endometriosis mice and found that the growth of lesions was restored, indicating that gut bacteria are involved in the progression of endometriosis lesions (Chadchan et al., 2019). Possible explanations for the effect of gut microbiota dysbiosis on endometriosis include the bacterial contamination theory, estrogen metabolism, cytokines and impaired immune function (Zizolfi et al., 2023). Long-term inflammation induced by EMS may also impair intestinal barrier function and further promote an imbalance in the intestinal microbiota and aggravate EMS inflammation, thus forming a vicious cycle (**Figure 2**). The gut microbiota may be used as a biomarker for the early diagnosis of EMS in the future (Huang et al., 2021b).

**Table 1:** Major studies investigating the association between endometriosis and microbial changes.

Design	Amount	Number of days	Result	Ref
Mouse case control	Inapplicability	42 days	In mice with endometriosis, the *Firmicutes/Bacteroidetes* ratio increased, *Bifidobacteria* also increased, and *Ruminococcus bromii* abundance decreased	(Yuan et al., 2018)
Mouse case control	Inapplicability	21 days	*Bacteroides* abundance increased, *Firmicutes* abundance decreased	(Chadchan et al., 2019)
Mouse case control	Inapplicability	Inapplicability	The diversity and abundance of the gut microbiota decreased	(Ni et al., 2020)
Case control	12 stage 3/4 EMS patients and 12 healthy controls	Inapplicability	The α diversity of intestinal microbiota in EMS group was lower and the ratio of *Firmicutes/Bacteroidetes* was higher	(Shan et al., 2021)
Case control	Women with endometriosis (N=66), each matched with three healthy controls (N=198)	Inapplicability	α and β diversity were higher in control group than in patients. The abundances of 12 bacteria belonging to the genera *Bacillus*, *Bacteroides*, *Clostridium* and *Coriobacillus* differed significantly between patients and controls.	(Svensson et al., 2021)
Pathological report	A woman diagnosed with endometriosis	Inapplicability	There were changes in the gut microbiota in EMS patients compared to those without the disease, with high levels of *Escherichia coli* and *Bifidobacteria* identified	(Bauşic et al., 2023)

### 4.1 Bacterial contamination theory

The human lower reproductive tract harbors a diverse microbiota, which can ascend to the upper reproductive tract through direct migration and potentially cause infections (Cheng et al., 2021). Khan et al. proposed that some Gram-negative bacteria, such as *E. coli*, infect the uterine wall after migrating up from the vagina to contaminate menstrual blood. Their study found that women with endometriosis had higher levels of *E. coli* in their menstrual blood than the control group (Khan et al., 2010). Combined with the theory of menstrual reflux, menstrual blood may flow from the fallopian tubes into the pelvic cavity (Halme et al., 1984). Bacterial lipopolysaccharide (LPS) is the main component of the cell wall of Gram-negative bacteria such as *E. coli*. LPS is constantly shed by living bacteria and is released when the bacteria die (Raetz and Whitfield, 2002). Khan et al. considered that *E. coli* contamination in her menstrual blood was a continuous source of LPS in the peritoneal cavity. Elevated LPS levels in peritoneal fluid may promote Toll-like receptor 4 (TLR4)-mediated progression of endometriosis (Khan et al., 2010). Another possible explanation for the residual accumulation of bacterial endotoxins in the pelvic environment is the transfer of *E. coli* or endotoxins from the intestine through intestinal cells and into the pelvic cavity (Deitch et al., 1989; Alexander et al., 1990). Dysbiosis of the intestinal microbiota can lead to an increase in Gram-negative bacteria, causing a large amount of LPS to enter the circulatory system inducing chronic inflammation throughout the body (He et al., 2019). Studies have shown that patients with endometriosis have elevated intestinal and serum LPS levels (Ni et al., 2020). Primary inflammation caused by LPS promotes the secretion of the NF-κB mediator in the peritoneal cavity through TLR4 present on macrophages, neutrophils, and other immune cells, followed by a cascade of reactions such as the production of significant levels of tumor necrosis factor-α (TNF-α) and interleukin-8 (IL-8), thus promoting the development of endometriosis (Khan et al., 2018; Jeljeli et al., 2020). Although this theory provides a new strategy for treating EMS by targeting bacterial endotoxin or TLR4 to suppress inflammation, it is currently not feasible for clinical application.

### 4.2 Estrogen metabolism

Another possible mechanism by which the gut microbiota is involved in the development of endometriosis is through the regulation of estrogen (Baker et al., 2017). Estrogen plays an important role in maintaining the development of the female reproductive system and is closely associated with the development of endometriosis (Jeon et al., 2016; Galvankar et al., 2017). Studies have found that estrogen can induce proliferative diseases such as endometriosis, endometrial cancer, and hysteromyoma by stimulating the proliferation of female genital epithelial cells (Xu et al., 2019; Somasundaram et al., 2020). Endometriosis has been associated with alterations in estrogen signaling (Laux-Biehlmann et al., 2015). Endometriotic women have a heightened proinflammatory and anti-apoptotic response to estradiol (Reis et al., 2013). This attributed to changes in nuclear estrogen receptor expression. Endometriotic lesions express higher levels of estrogen receptor-β (ER-β), whose signaling promotes lesion growth by inhibiting TNF-α-induced apoptosis, activating an inflammasome which increases IL-1β, and enhancing cellular adhesion and proliferation (Han et al., 2015). The gut microbiota plays an important role in estrogen metabolism, and the gene pool in the gut microbiota capable of metabolizing estrogen is called the “estrogen group” (Salliss et al., 2021). It has been observed that the use of antibiotics leads to a decrease in estrogen levels (Chadchan et al., 2019). Estrogen metabolism takes place mainly in the liver. Estrogens are inactivated in the liver into less active estrone and estriol and bind to glucuronic acid to promote their excretion in bile, urine, and feces (Adlercreutz, 1974). However, beta-glucuronidase secreted by gut microbes can metabolize estrogen from the bound form to the unbound form (Ervin et al., 2019; Sui et al., 2021). Free estrogens can be absorbed into the circulatory system, resulting in increased levels of free estrogens, and can be transported through the blood to distal mucosal sites, such as the endometrium. Estrogen binds to estrogen receptors, which are subsequently internalized and function as transcription factors promoting the transcription of genes involved in cellular growth (Dabek et al., 2008; Baker et al., 2017; Salliss et al., 2021). Studies have shown that multiple genera in the gut microbiota, including *Bifidobacteria*, *Bacteroidetes*, *Escherichia coli* and *Lactobacillus*, can encode β-glucuronidase (Leonardi et al., 2020). When the intestinal flora is dysregulated, the flora producing β-glucuronidase in the gut increases, leading to elevated circulating estrogen levels and promoting the proliferation of ectopic endometrial lesions (Baker et al., 2017). Some studies have found that the levels of *Bifidobacteria* and *Escherichia* in the endometriosis group are higher than those in the control group (Yuan et al., 2018). In addition, elevated levels of β-glucuronidase expression were observed in endometriosis lesions compared to normal endometrium (Wei et al., 2023). Metabolites produced by the gut microbiota also affect estrogen metabolism and affect endometriosis. For example, short-chain fatty acids produced by some bacteria can affect estrogen metabolism by regulating the expression of estrogen-metabolizing enzymes in the liver and other tissues, affecting estrogen levels and hormone balance in endometriosis (Ervin et al., 2019). In summary, the gut microbiota can lead to increased circulating estrogen levels, which can promote the development of endometriosis (Qi et al., 2021). However, whether specific gut microbiota constituents stimulate β-glucuronidase production and whether the gut microbiota has synergistic effects on the pathogenesis of EMS remain to be studied further.

### 4.3 Immunity and inflammation

Immunity and inflammation play an integral role in the pathogenesis of endometriosis. The results of the sustained growth of endometrial lesions in ovariectomized animals suggest that the innate immune system in the pelvic environment can also influence the growth of endometriosis lesions in addition to the influence of sex hormones (Novella-Maestre et al., 2012). One study demonstrated that the number of macrophages, monocytes, and cytokines in the peritoneal fluid of women with endometriosis was increased, and the levels of the pro-inflammatory factors IL-1β, IL-18 and TGF-β are increased (Zhou et al., 2019; Agostinis et al., 2020). Increased numbers of macrophages help to secrete proinflammatory cytokines, such as transforming growth factor-β1 (TGF-β1), IL-8, IL-6, TNF-α, and IL-1β (Jeljeli et al., 2020). These cytokines are expressed mainly by macrophages in the peritoneal cavity through increasing the activation of the NF-κB pathway. A local cytokine surge can further promote the recruitment of immune cells and the secretion of additional cytokines, and stimulate the production of vascular endothelial growth factor (VEGF), which is conducive to angiogenesis (Becker and D'Amato, 2007; Symons et al., 2018). VEGF is a major regulator of vascularization and can induce endometrial cell proliferation (Young et al., 2015). In addition, when the inflammatory activity in the peritoneal fluid decreases, the level of the anti-inflammatory factor IL-37 increases. When inflammatory substances cannot be removed, an inflammatory response may occur, which spreads throughout the body, with long-term effects on the immune system (Symons et al., 2018; Zhou et al., 2019).

At present, many studies have confirmed the close relationship between gut microbiota and inflammation (Buford, 2017; Shi et al., 2017; Clemente et al., 2018; Brandsma et al., 2019). Jiang et al. hypothesized that changes in the gut and genital tract microbiota would lead to changes in the immune response. Gut microbiota dysbiosis destroys normal immune function and leads to the elevation of proinflammatory cytokines, which impairs immune surveillance in the pelvic region and changes the immune cell spectrum, creating an inflammatory microenvironment to promote the persistence of endometriosis (Jiang et al., 2021). Yuan et al. showed that the gut microbiota may interact with cytokines abnormally expressed in endometriotic lesions, peritoneal fluid and peripheral blood (Yuan et al., 2018). The effect of gut microbiota on host inflammatory response is mediated by a variety of mechanisms. Dysregulation of the gut microbiome can digest the protective mucus layer of the gut and interact directly with intestinal cells, leading to increased local and systemic inflammation (Blander et al., 2017). For example, segmented filamentous bacteria can directly stimulate T lymphocytes to differentiate into helper T lymphocytes (Th17) to drive autoimmune arthritis (Wu et al., 2010). Many studies have shown that Th17 cells and their cytokines are significantly increased in the peritoneal fluid of women with endometriosis (Gogacz et al., 2016; Tarokh et al., 2019). As previously mentioned, intestinal microbiota imbalance can also lead to LPS leakage by increasing intestinal permeability, which activates the innate immune system through TLR4 and increases the expression of pro-inflammatory cytokines, thus promoting the occurrence and development of endometriosis (Khan et al., 2010; Bruner-Tran et al., 2013). In addition, short-chain fatty acids act as a medium of communication between the gut microbiome and the immune system, serving as an energy source for intestinal cells or being transported into the blood, not only acting in the gut, but also regulating immune responses in distant tissues (Parada Venegas et al., 2019; Chadchan et al., 2021). These are described in detail later in Chapter 6. However, the molecular mechanisms that explain the microbiome’s interaction with immune inflammation are not fully understood, and more research is needed to explore the complex role of the gut microbiome in the multi-factorial etiology of EMS, including impaired cytokine and immune function.

## 5 Relationship between the gut microbiota and SCFAs interaction

### 5.1 The gut microbiota regulates SCFAs metabolism in the host

The literature clearly highlights that gut microbes play a key role in the metabolism of short-chain fatty acids (Mirzaei et al., 2022). In the gut, SCFAs are the main beneficial metabolites produced by gut microbes through the metabolism of indigestible dietary fiber. The most abundant SCFA is acetate, which is produced mainly by *Bacteroides*, one of the largest flora in the gut. Acetate can be produced from pyruvate *via* the acetyl-CoA or Wood-Ljungdahl pathways (Ragsdale and Pierce, 2008). Another major SCFA, propionate, is produced by a few dominant bacterial genera, such as *Ruminococcus bromii, Veillonella parvula* and *Bacteroides* (van der Hee and Wells, 2021). Propionate is mainly produced from succinate *via* the succinate pathway or from lactate *via* the acrylate pathway (Koh et al., 2016). The third major SCFA, butyrate, is mostly produced by *Firmicutes*, such as *Faecalibacterium*, *Eubacterium rectale* and *Eubacterium hallii* (Vital et al., 2014). Butyrate is formed from acetyl-CoA and butyryl-CoA (Koh et al., 2016). There are metabolic linkages between different types of bacteria. For example, acetate produced by *Bacteroides* can be utilized by *Firmicutes* to produce butyrate. The diversity and quantity of host gut microbes are the main factors affecting the production of SCFAs. One randomized trial revealed that resistant starch was able to regulate the gut microbiome, such as increased *Bifidobacteria*, altered *Firmicutes* to bacteria ratio, and observed an increase in the relative abundance of butyrate (Alfa et al., 2018). In addition, a new study examined whether *Bifidobacteria* can synthesize SCFAs *in vivo* by LC−MS/MS and revealed that *Bifidobacteria* mice and mice without a specific pathogen exhibit high concentrations of acetic acid and propionate. *Bifidobacteria* colonization and changes in the gut microbiota can affect the abundance of intestinal short-chain fatty acids (Horvath et al., 2022). The above studies showed that diet and the gut microbiota are the influencing factors of SCFAs metabolism.

### 5.2 SCFAs inhibit the growth of harmful bacteria

While gut microbes regulate the metabolism of short-chain fatty acids, SCFAs produced in the gut can inhibit the growth of harmful intestinal bacteria, which play an important role in maintaining the stability of the gut microbiota. Studies have shown that SCFAs can remodel antibiotic-induced disordered gut microbiota by increasing the number of beneficial bacteria and reducing the number of harmful bacteria. After the administration of SCFAs, the relative abundance of *Firmicutes* and *Bacteroides* in septic mice increased significantly, and the number of *Enterobacteriaceae*, such as *Proteobacteria* and *Escherichia Shigella*, was significantly reduced and returned to levels comparable to those in healthy mice (Lou et al., 2022). Oral administration of butyrate to mice can significantly enhance the antibacterial activity of colonic macrophages, for example, by conferring better killing effects against *Salmonella enterica* and *Citrobacterrodentium* (Flemming, 2019). SCFAs inhibit the growth of harmful bacteria mainly by releasing H^+^, reducing the intestinal pH, competing for energy, blocking the biosynthesis of harmful bacteria, and forming antibacterial peptides to achieve intestinal microecological balance (**Table 2**). In the intestine, some SCFAs are protonated by the carboxyl and phosphate groups of lipopolysaccharides on the bacterial outer membrane, destroying the integrity of the bacterial outer membrane; in addition, another part of SCFAs dissociates in bacterial cells to produce large amounts of RCOO^-^ and H^+^. H^+^ disrupts intracellular pH homeostasis; RCOO^-^ increases intracellular osmotic pressure, forcing these cells to excrete precursors or accessory substances necessary for their growth to balance osmotic pressure, thus inhibiting the growth of harmful bacteria (Ma et al., 2022). Moreover, the continuous accumulation of RCOO^-^ changes the homeostatic environment in bacterial cells, thereby interfering with a series of physiological and biochemical characteristics inside bacteria and thereby blocking the biosynthesis of harmful bacteria. In addition, SCFAs trigger host cells to produce certain antibacterial peptides by activating and regulating the host immune system, which in turn achieves bacterial inhibition by lysing the bacterial cell membrane, resulting in leakage of the contents and death of the bacteria (Raqib et al., 2006; Jakubowski, 2019).

**Table 2:** Short-chain fatty acids inhibit harmful intestinal bacteria.

Types of SCFAs	Bacterial species	Machine	Ref
Colon SCFAs	*Escherichia coli*	PH-dependent	(Zhang et al., 2020)
SCFAs	*Streptococcus gordonii*	The expression of comD and comE was inhibited, and the formation of bacterial biofilm was inhibited	(Park et al., 2021)
SCFAs	*Salmonella*	Decreased motility, inhibited biofilm formation and upregulated virulence gene expression	(Lamas et al., 2019)
SCFAs	*Candida albicans*	Inhibit the growth of *Candida albicans*, the development of embryonic tubes, mycelia and biofilms	(Guinan et al., 2019)
propionate	*Oral Streptococci*	Enhance methionine biosynthesis	(Park et al., 2022)
propionate	*Staphylococcus aureus*	Reduces the intracellular pH of bacteria	(Jeong et al., 2019)
SCFAs	*Salmonella*	Induce the expression of antimicrobial host defense peptide gene	(Sunkara et al., 2011)
butyrate	*Helicobacter pylori*	Destructive effect on cell membranes	(Yonezawa et al., 2012)

## 6 Inhibition of EMS by SCFAs relevant mechanisms

In recent years, with increasing research on the interaction between gut microbiota and EMS, it has been proposed that gut microbiota metabolites (such as short-chain fatty acids and bile acids) can affect EMS occurrence and development (Chadchan et al., 2021; Li et al., 2022; Chadchan et al., 2023). SCFAs, key signaling molecules involved in the interaction between the gut microbiota and host, mediate the NF-κB signaling pathway and MAPK signaling mainly by activating G protein-coupled receptors (GPCRs) and inhibiting histone deacetylase (HDAC). Therefore, SCFAs play an anti-inflammatory and regulatory role in immune cells, inhibiting the survival of endometriotic cells and the growth of lesions, thereby exerting an inhibitory effect on EMS (Chadchan et al., 2021, 2023).

**Figure 3:**
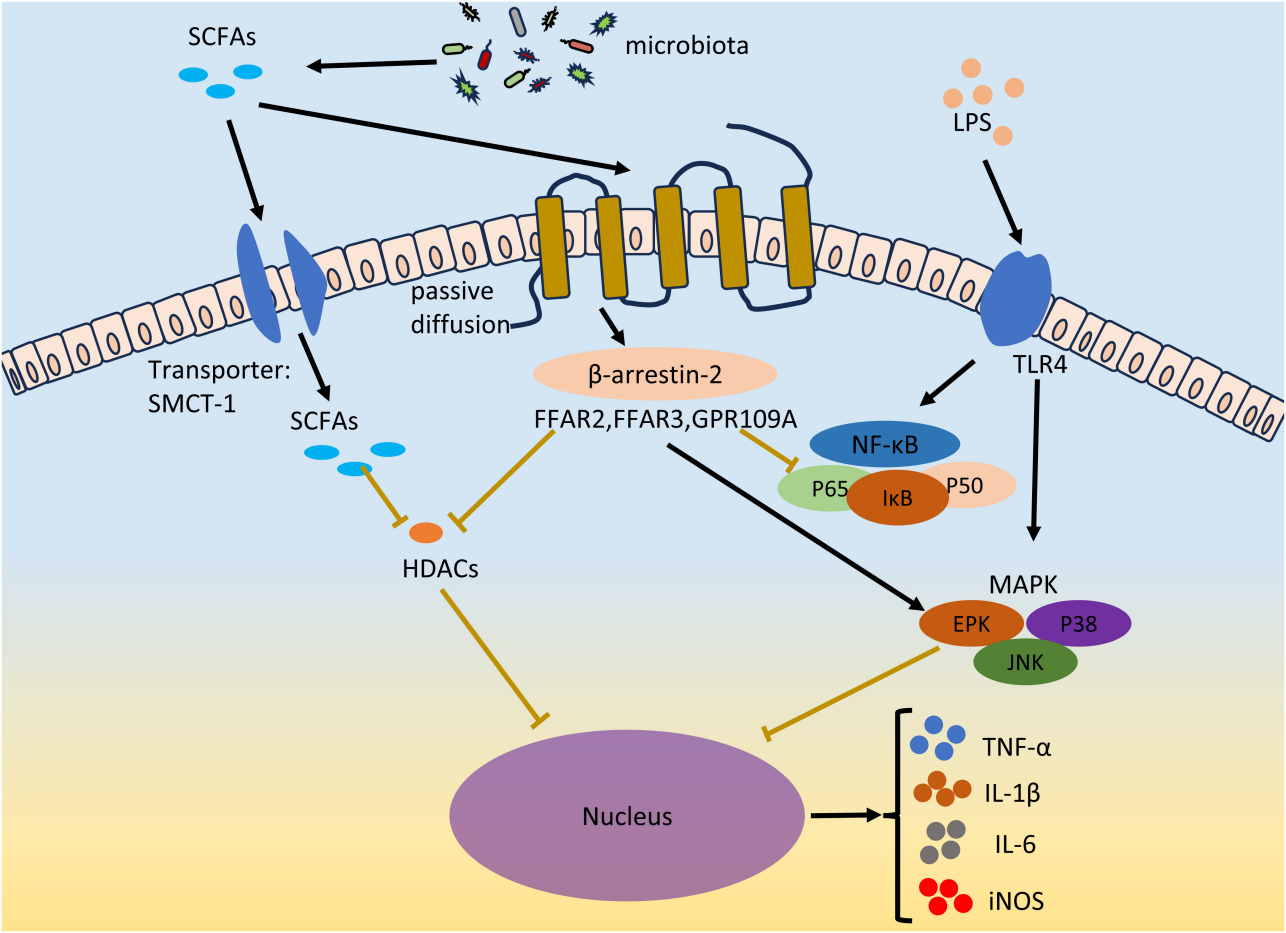
SCFAs inhibits EMS inflammation. LPS acts on TLR4 on immune cells, and promotes the secretion of pro-inflammatory factors TNF-α, IL-1β and IL-6 and the expression of iNOS by activating two inflammatory signaling pathways, NF-κB and MAPK. Gut microbiota derived SCFAs inhibit LPS-induced inflammation by activating GPCRs receptors and inhibiting HDACs in both ways. SCFAs enters through passive diffusion and then reduces NF-κB levels and the amount of p65 and p50 subunits of NF-κB entering the nucleus via the β-arrestin-2 signaling pathway or FFAR2, FFAR3, and GPR109A receptors. SCFAs regulates MAPK signaling pathways, including ERK, JNK and P38MAPK signal transduction pathways. SCFAs can inhibit HDACs through GPCRs, or directly inhibit HDACs through the entry of the transporter protein (SMCT-1) into cells. The secretion of TNF-α, IL-1β and IL-6 and the expression of iNOS were inhibited. The black lines indicate activation and the brown lines indicate inhibition.

The inflammatory environment and local and systemic immune cell dysfunction are characteristics of EMS (Riccio et al., 2018; Symons et al., 2018; Zhang et al., 2018; Viganò et al., 2020). The levels of proinflammatory cytokines (such as TNF-α, IL-1β, IL-6, and IL-17A) in ectopic lesions in patients with endometriosis increase to promote inflammation and the development of EMS (Ramírez-Pavez et al., 2021; Chen et al., 2023). The LPS concentration is a diagnostic marker of inflammatory diseases. LPSs act on TLRs on macrophages or neutrophils to promote the secretion of the proinflammatory cytokines TNF-α, IL-1β and IL-6, resulting in inflammation (Park and Lee, 2013). The two main inflammation-related signaling pathways in cells, the NF-κB and MAPK pathways, were activated after TLR activation. Two subunits of NF-κB, P65 and P50, are acetylated and translocated from the cytoplasm to the nucleus to promote the secretion of proinflammatory cytokines (Vallabhapurapu and Karin, 2009). Currently, NF-κB has been found to mediate a variety of cytokines (TNF-α, IL-1β, IL-2, IL-6, IL-8, and IL-12) and chemokines (MIP-2 and MCP-1) transcription (Liu and Malik, 2006). The MAPK signaling pathway is involved in the regulation of various key functions in the body and plays an important role in cell proliferation, differentiation and apoptosis. Phosphorylated mitogen-activated protein kinase (MAPK) regulates the ERK, JNK, and P38MAPK signal transduction pathways; gene transcription; secretion of the proinflammatory cytokines TNF-α and IL-6; and the expression of iNOS and nitric oxide (NO) (Arthur and Ley, 2013). Abnormal activation of the MAPK cascade can enhance the proliferation and survival of endometrial cells in EMS patients (Yotova et al., 2011). *In vivo* administration of a p38 MAPK inhibitor can significantly inhibit endometriotic lesions and reduce the levels of proinflammatory factors (IL-1β and TNF-α) and chemokines (MMP2 and MMP9) in peritoneal fluid (Zhou et al., 2010). Many studies have shown that LPS-mediated proinflammatory cytokines (such as IL-6) can be inhibited by SCFAs and that SCFAs can suppress LPS-induced inflammation by activating GPCR receptors and inhibiting HDACs (Vinolo et al., 2011) (**Figure 3**).

### 6.1 Activation of GPCRs by SCFAs

SCFAs can exert anti-inflammatory effects on many cell types through the activation of G protein-coupled receptors, thereby inhibiting the development of EMS. Among them, GPR43 (also known as FFAR2), GPR41 (also known as FFAR3) and GPR109A are the most important receptors for SCFAs in the GPCR family. FFAR2 is widely expressed in neutrophils, monocytes, intestinal Treg cells, eosinophils and other immune cells (Smith et al., 2013). The FFAR2 receptor reduces NF-κB levels through the β-arrestin-2 signaling pathway and reduces the number of NF-κB subunits, p65 and p50, in the nucleus, thereby inhibiting the transcription of proinflammatory cytokines (IL-1β and IL-6) (Lee et al., 2013). SCFAs can inhibit the expression of IL-6, IL-1β and TNF-α through FFAR2, thereby exerting their anti-inflammatory effects (Nakajima et al., 2017; Pirozzi et al., 2018; Mizuta et al., 2019). Consistent with its function in FFAR2, FFAR3 also plays important roles in inflammation. C3 can inhibit the expression of IL-4, IL-5 and IL-17A through FFAR3, and C4 can inhibit the expression of nitric oxide synthase (iNOS), TNF-α, IL-6 and monocyte chemoattractant protein-1 (MCP-1) through FFAR3 (Li et al., 2018). In contrast to FFAR2 and FFAR3, GPR109A is activated by longer SCFAs, mainly C4 (Offermanns, 2017). Li et al. reported that GPR109A exerts an anti-inflammatory effect by inhibiting the Akt/mTOR signaling pathway in MIN6 pancreatic β cells (Priyadarshini et al., 2018).

### 6.2 Inhibition of HDACs by SCFAs

As a protease, histone deacetylases (HDACs) play important roles in the regulation of gene expression and the modification of chromosome structure. SCFAs are considered to be natural inhibitors of HDACs. Among all short-chain fatty acids, butyrate is the most effective inhibitor of HDAC activity. SCFAs may directly act on HDACs to enter cells through transporters or indirectly act on HDACs through GPCR activation. For example, C4 inhibits LPS-induced production of nitric oxide and inflammatory cytokines (such as IL-6 and IL-12) but directly inhibits HDACs and is unrelated to GPCRs (Chang et al., 2014). The authors of this study believe that C4 acts directly on HDACs. This is because SCFAs can be coupled to monocarboxylate transporter 1 (SMCT-1) through sodium transporter proteins rather than through membrane receptors, thereby entering cells, occupying the active sites of HDACs and inhibiting HDACs (Sun et al., 2017). In another report, the authors found that activation of FFAR3 could repress histone acetylation in a Chinese hamster ovary cell line through the inhibition of HDACs (Wu et al., 2012). In addition, FFAR3, FFAR2 and GPR109 may all be involved in the inhibition of HDACs by SCFAs. However, how SCFAs inhibit HDAC activity through GPCRs or directly has not been determined, and additional studies are needed to determine the underlying mechanisms involved.

### 6.3 Regulation of immune cells by SCFAs

SCFAs produced by the gut microbiota are transported throughout the body and can maintain systemic homeostasis by inhibiting inflammatory immune cells and promoting the differentiation of immune cells. We describe in detail how SCFAs regulate various immune cells, including macrophages (Macs), neutrophils, Tregs, and dendritic cells (DCs), thereby suppressing EMS-related inflammation.

#### 6.3.1 Macs

The phenotype of Macs changes according to the surrounding microenvironment, and their function also changes with the phenotype. Macrophages are usually divided into two phenotypes: inflammatory macrophages (iMacs) and tolerant macrophages (tMacs). IMacs can participate in the inflammatory immune response, while tMacs suppress inflammation and maintain homeostasis by producing large amounts of anti-inflammatory factors, such as IL-10 and TGF-β. Macrophages play a major role in the development and expansion of EMS. Endometrial lesions are composed mainly of anti-inflammatory M2-polarized macrophages (Artemova et al., 2021). SCFAs can significantly reduce HDAC mRNA expression in Macs (Eslick et al., 2022). SCFAs, especially butyrate, inhibit the activation of Macs by negatively regulating NLRP3-mediated inflammatory signaling pathways (Shao et al., 2021). Furthermore, only butyrate can reprogram Macs metabolism to oxidative phosphorylation, converting Macs to an anti-inflammatory resistant phenotype (Scott et al., 2018).

#### 6.3.2 Neutrophils

Neutrophils play an important role in the progression of EMS, and together with other leukocytes, they are involved in the regulation of EMS, including inflammation and repair (Huang et al., 2022). Several studies have reported increased neutrophil concentrations in plasma and peritoneal fluid during EMS. Moreover, the phagocytic ability of neutrophils is significantly reduced (Lukács et al., 2022). Studies have shown that SCFAs can enhance the phagocytosis of neutrophils at sites of inflammation by activating the GPR43 receptor (Vinolo et al., 2009). In addition, butyrate and propionate can also suppress the secretion of the proinflammatory factor TNFα and the activity of NF-κB and upregulate the expression of the anti-inflammatory factor IL-10 in LPS-activated monocytes and neutrophils through the inhibition of HDACs (Aoyama et al., 2010).

#### 6.3.3 Tregs

Tregs play a key role in maintaining EMS immune homeostasis. The differentiation and function of Tregs can be regulated by SCFAs. Treg cells are present in large quantities in endometriotic lesions (Abramiuk et al., 2022). In some studies, the complete elimination of Tregs in EMS mice led to an increased volume of ectopic lesions (Tanaka et al., 2017). However, the role of Tregs in the progression of endometriosis has not been determined. SCFAs can promote the release of IL-10 from Treg cells through FFAR2. The main mechanism by which SCFAs regulate Tregs is to mediate signal transduction by activating GPCRs on the surface of target cells and inhibit HDACs to regulate gene expression (Smith et al., 2013). Butyrate increases histone acetylation to promote the differentiation of Tregs or promote the differentiation of Tregs by regulating dendritic cells (DCs) (Chung et al., 2009; Furusawa et al., 2013). In addition, SCFAs promote IL-10 production in Th1 and Th17 cells (Park et al., 2015).

#### 6.3.4 DCs

DCs are antigen-presenting cells necessary for the activation of naive T cells. After DCs acquire tolerance, they participate in the elimination of the immune response. Furthermore, tolerable DCs (tDCs) mainly contribute to Treg differentiation and homeostasis. Butyrate and propionate inhibit the activation of bone marrow-derived DCs (BMDCs) by inhibiting the LPS-mediated expression of costimulatory molecules (such as CD40) and the production of cytokines such as IL-6 and IL-12 (Nastasi et al., 2015).

## 7 Treatment strategies

**Figure 4:**
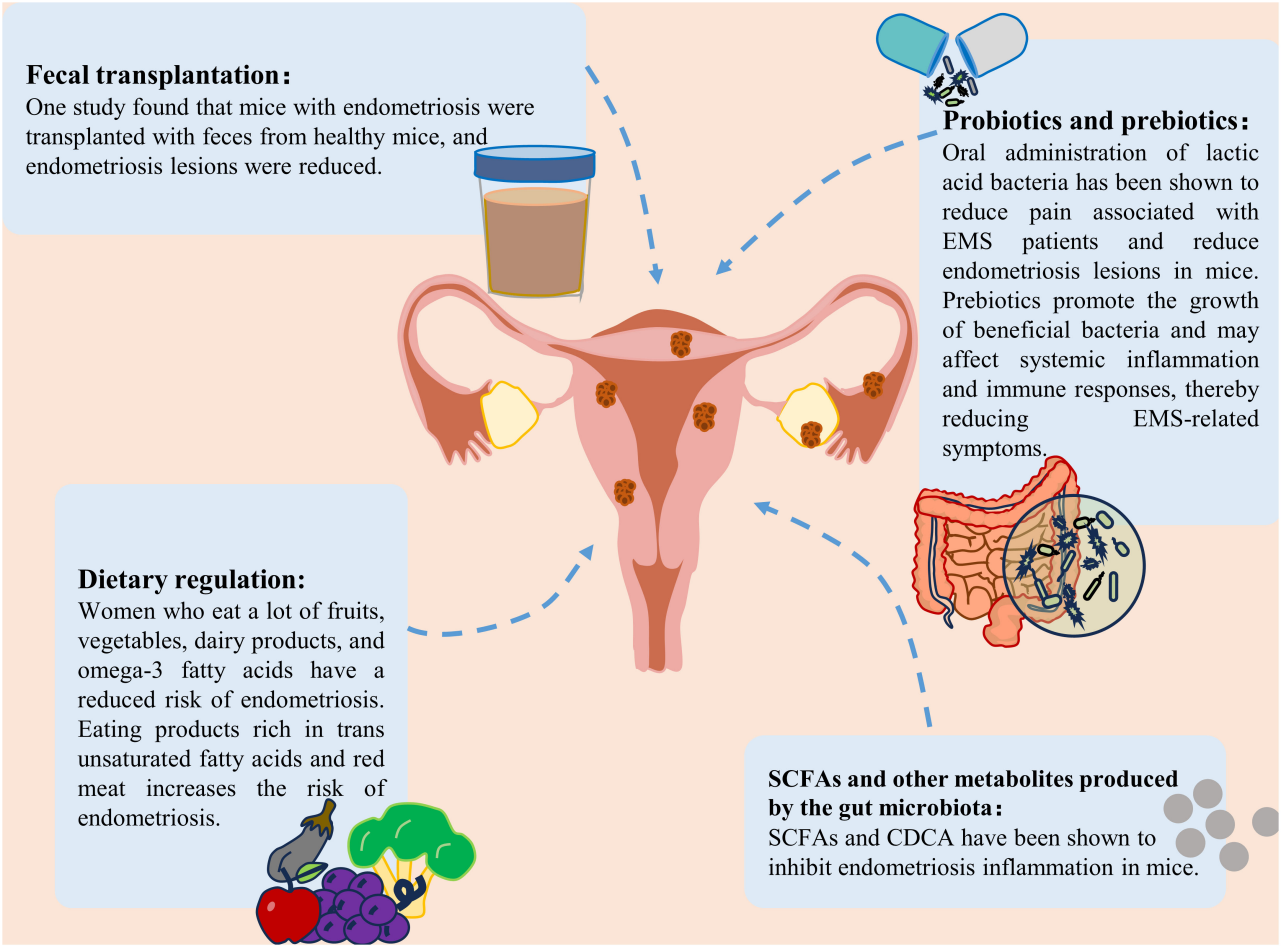
Treatment strategies for endometriosis. Treatment strategies for endometriosis include the use of probiotics and prebiotics, fecal bacterial transplantation, gut microbiota metabolite supplementation and dietary modification.

At present, the clinical treatments for EMS are mainly surgical treatment, drug treatment, or a combination of the two. It is necessary to find safe and effective methods to prevent and treat EMS (3). In recent years, an increasing number of researchers have shifted their focus to the gut microbiota and its metabolites. An imbalance in the gut microbiota strongly impacts the occurrence and development of EMS. Regulating the gut microbiota to restore it to a normal state could open up new avenues for the treatment of EMS. We reviewed the current potential interventions targeting the gut microbiota and SCFAs for the treatment of EMS, including probiotic and prebiotics, fecal transplantation (FMT), gut microbiota metabolite therapy and diet regulation, which may provide new ideas into the clinical treatment of EMS (**Figure 4**).

### 7.1 Probiotics and prebiotics

Probiotics and prebiotics are two components of new therapies based on the gut microbiota. Probiotic intervention is an effective method for treating EMS. The potential effects of probiotics on immune regulation, inflammation and intestinal flora imbalance are all associated with the development of EMS (Bodke and Jogdand, 2022; Hashem and Gonzalez-Bulnes, 2022). Oral *lactic acid bacteria* have been shown to reduce pain associated with endometriosis (Khodaverdi et al., 2019). It also reduces endometriotic lesions in mice by enhancing NK cell activity and increasing the IL-12 concentration (Itoh et al., 2011). Dysbiota and endometriosis-related inflammation lead to impaired NK cell activity, and probiotic treatment reversed this immune dysregulation. In a rat model, probiotic treatment was also found to inhibit the growth of endometriosis (Uchida and Kobayashi, 2013). In addition, as mentioned previously, SCFAs can inhibit endometriosis. *In vitro* and *in vivo* studies have confirmed the positive effect of probiotics on increasing short-chain fatty acids (Markowiak-Kopeć and Śliżewska, 2020). The use of different types of probiotics has been found to increase SCFAs levels in patients with different diseases (such as colorectal cancer and respiratory diseases) (Hou et al., 2022; Williams et al., 2022). It was speculated that probiotics may also inhibit EMS by increasing the production of SCFAs.

To increase the effectiveness of probiotics, they can be combined with prebiotics. Although studies specifically focusing on the effects of prebiotics on endometriosis are limited, prebiotics can promote the growth of beneficial bacteria, reduce the translocation of harmful bacteria and their products into the blood, and help maintain the ecological balance of the intestinal microbiota. Subsequently, it may affect systemic inflammation and the immune response, thereby affecting EMS-associated symptoms (You et al., 2022). In addition, prebiotics are widely used to increase SCFAs levels (Gibson et al., 2017). One study showed that the use of high doses of arabinoxylan oligosaccharides could increase butyrate, acetate, propionate, and total SCFAs (François et al., 2012). Therefore, prebiotics may have potential benefits for treating endometriosis. Although probiotics and prebiotics have shown good potential in treating EMS, there is still a lack of guidelines outlining or supporting the standard use of probiotics and prebiotics in EMS management, and additional studies are needed to evaluate the effects of probiotics and prebiotics on EMS in the future.

### 7.2 Fecal microbe transplantation

FMT involves the transfer of feces from healthy people to the gastrointestinal tract of patients with imbalanced flora to reconstruct the gut microbiota and treat related diseases (Nigam et al., 2022). Currently, FMT has been applied to treat many diseases, such as gastrointestinal diseases, neuropsychiatric diseases, and blood diseases (Kim and Gluck, 2019). In addition, FMT has become a mature therapy for recurrent *Clostridium difficile* infection (Cammarota et al., 2015). Recently, studies have shown that FMT can be used to treat female reproductive tract diseases (Quaranta et al., 2019; Huang et al., 2021a). A study by Chadchan et al. revealed that after endometriosis mice were transplanted with feces from healthy mice, the number of uterine lesions decreased (Chadchan et al., 2021, 2023). Ni et al. recently reported similar findings that ZO-2 expression was reduced after the administration of feces from endometriotic mice to healthy mice (Ni et al., 2021). These preliminary studies highlighted the potential of FMT to relieve EMS through the reinforcement of barrier integrity. In addition, several pieces of evidence suggest that an increase in SCFAs may be a key determinant in the treatment of different diseases by FMT (Chen et al., 2019; El-Salhy et al., 2021; Xu et al., 2023). Taken together, these findings indicate that FMT might be a potential treatment for EMS. However, this strategy is limited by the presence of unknown microorganisms and pathogenic bacteria in the transplanted feces.

### 7.3 SCFAs and other metabolites produced by the gut microbiota

One study revealed that SCFAs play an important role in maintaining the intestinal barrier and suppressing immune function (Rekha et al., 2022). Among them, butyric acid played a particularly significant role. Butyrate can also suppress the immune response caused by intestinal microbial imbalance (Ma et al., 2020). A recent study showed that gut microbiota-derived butyrate could inhibit endometriosis in mice through the regulation of G protein-coupled receptors (Chadchan et al., 2021). In addition, another type of microbiota metabolite that has therapeutic effects on EMS is secondary bile acids. A study on the correlation between fecal metabolomics and the gut microbiota in endometriosis mice revealed that bile acid and chenodeoxycholic acid (CDCA) levels were increased in the intestines of endometriosis mice (Ni et al., 2020). The above studies showed that changes in the metabolite levels of the gut microbiota may play an important role in the development of EMS. Although several metabolites of the gut microbiota are maypotentially useful for treating EMS, the functional diversity of these metabolites needs to be overcome to design effective treatment strategies.

### 7.4 Dietary adjustment

Dietary intervention may lead to different changes in the gut microbiota and SCFAs levels. Diet provides substrates that enable the manipulation of microbial composition. For example, the Mediterranean diet can increase the availability of substrates available for specific bacteria, thereby increasing the formation of SCFAs (Wagenaar et al., 2021; Yalçın Bahat et al., 2022). A fiber-rich diet can increase the levels of SCFAs, especially acetate and butyrate (So et al., 2018). Rinninella et al. evaluated the use of a ketogenic diet and reported that the total number and abundance of several beneficial bacteria and bacteria, were reduced. A ketogenic diet can significantly affect the characteristics of the gut microbiota and may lead to a reduction in short-chain fatty acids (Rew et al., 2022; Rinninella et al., 2023). A plant-based diet has been shown to lead to the dominance of SCFAs-producing *Firmicutes*, while an animal-based diet increases the number of *Bacteroides* (Tomova et al., 2019). Recently, a large number of studies have shown that diet is a potential risk factor for endometriosis (Jurkiewicz-Przondziono et al., 2017). Women who consume large amounts of fruits, vegetables, dairy products, or omega-3 fatty acids (PUFAs) have a reduced risk of developing endometriosis (Samaneh et al., 2019). A high intake of omega-3 polyunsaturated fatty acids can reduce the risk of endometriosis in women, which may be attributed to potential changes in the gut microbiota (Watson et al., 2018). A diet rich in ω-3 polyunsaturated fatty acids can reduce inflammation in a mouse model of endometriosis, as indicated by reduced TNF-α and IL-6 inflammatory marker levels (Pereira et al., 2019). In addition, some studies have shown that linolenic acid (ALA) can reduce inflammation by inhibiting the accumulation of prostaglandin E2 (PGE2) and nitrite. ALA can also reduce LPS levels and improve the abdominal inflammatory environment in endometriosis mice (Ni et al., 2021). Moreover, it can inhibit the inflammatory response of M1 macrophages (Pauls et al., 2018). However, the consumption of products and red meat rich in trans-unsaturated fatty acids can increase the risk of endometriosis (Marcinkowska and Górnicka, 2023). In conclusion, reasonable diet adjustment may help prevent EMS.

In summary, the gut microbiota and SCFAs can be modulated by probiotics and prebiotics, fecal microbiota transplantation, gut microbiota metabolites, or dietary regimens. These methods can greatly increase the SCFAs produced in the colon, inhibiting EMS. However, the clinical efficacy of these methods needs further study.

## 8 Conclusions

In conclusion, gut microbes and their metabolite SCFAs can regulate the development of EMS. A gut microbiota imbalance promotes the development of EMS through mechanisms such as bacterial contamination, estrogen metabolism, cytokine production, and impaired immune function. The normal gut microbiota inhibits the development of EMS by increasing the production of SCFAs in the host. The specific underlying mechanisms include the inhibition of HDACs and the activation of GPCRs. In addition, this review summarizes the current potential interventions targeting the gut microbiota and SCFAs for the treatment of EMS, including probiotics and prebiotics, FMT, gut microbiota metabolites, and dietary regulation, which may be new directions in the clinical treatment of EMS. Provide new thinking.

Diagnostic delay is one of the main difficulties faced by EMSs. Studying the different characteristics of the microbiota and its metabolites in EMS patients will aid in noninvasive diagnosis, reduce the incidence of delayed diagnosis, and enable prevention. Notably, prevention is more important than treatment. At present, a large number of studies have confirmed the function of the gut microbiota in EMS. In recent years, studies have also reported the important role of microbiota metabolites (especially SCFAs and bile acids) in the pathogenesis of EMS. However, the treatment of EMS based on this therapy is still at the experimental research stage and has not yet been clinically applied. The experimental data may be affected by various factors, including race, diet, or specimen quality, and further research is needed to address these issues. Nevertheless, we should focus on the effect of the gut microbiota-SCFAs-EMS axis on the EMS. Understanding the key microbiota-derived metabolites associated with EMS is important for the future design of microbiome-based therapeutics. In addition, with the advancement of molecular techniques and at the genetic level, new biomarkers are expected to be obtained, and new treatment options are expected to be developed in the future; however, additional studies are needed.

## Author contributions

ML: Writing – original draft, Writing – review & editing. RP: Writing – original draft, Writing – review & editing. CT: Writing – original draft, Writing – review & editing. JS: Conceptualization, Writing – review & editing. JM: Visualization, Writing – review & editing. RS: Writing – review & editing. XQ: Writing – review & editing, Conceptualization. RZ: Funding acquisition, Writing – review & editing. HG: Supervision, Writing – review & editing.
